# Association between serum globulin and cognitive impairment in older American adults

**DOI:** 10.3389/fpubh.2023.1193993

**Published:** 2023-08-21

**Authors:** Jian Huang, Rong Li, Hao Zhu, Dong Huang, Weiwang Li, Jing Wang, Zhirong Liu

**Affiliations:** ^1^Department of Neurology, Xijing Hospital, Airforce Military Medical University, Xi'an, Shaanxi, China; ^2^Department of Nephrology, Xijing Hospital, Airforce Military Medical University, Xi'an, Shaanxi, China; ^3^Department of Neurology, Xianyang First People's Hospital, Xianyang, Shaanxi, China; ^4^Department of Neurology, The Second People's Hospital of Shaanxi Province, Xi'an, Shaanxi, China; ^5^Department of Neurology, Xi'an Daxing Hospital, Xi'an, Shaanxi, China; ^6^Department of Neurology, Xi'an First Hospital, Xi'an, Shaanxi, China

**Keywords:** serum globulin, cognitive impairment, Consortium to Establish a Registry for Alzheimer’s disease (CERAD), animal fluency (AF), Digit Symbol Substitution Test (DSST), smooth curve fit, generalized additive models (GAM)

## Abstract

**Background and aims:**

Cognitive impairment is on the rise around the world, with profound economic and social consequences. Serum globulin, a marker of liver function, may also play a role in cognitive function. Unfortunately, no consistent conclusion exists regarding the association between serum globulin and cognitive function.

**Methods:**

Data from the 2011 to 2014 National Health and Nutrition Examination Survey were used to assess the association between serum globulin and cognitive impairment. Cognitive function was assessed by three tests: Consortium to Establish a Registry for Alzheimer’s Disease (CERAD), Animal Fluency (AF), and Digit Symbol Substitution Test (DSST). Furthermore, the breakthrough point of cognitive impairment correlated with CERAD < 5, AF < 14, and DSST < 34. A weighted multiple logistics regression model was used to verify the association between serum globulin and cognitive impairment. Generalized additive models (GAMs) and a smooth curve fit (penalty spline method) were used to determine a non-linear relationship between serum globulin and cognitive impairment. Finally, subgroup analysis and interaction tests were conducted to further verify the association between serum globulin and cognitive impairment.

**Results:**

Data from 2,768 participants aged ≥60 (in accordance with the study design) were collected for the final analysis. Data suggested that serum globulin levels were associated with an elevated cognitive impairment based on the AF [full adjustment, OR = 1.05, 95% *CI:* 1.01–1.08] and DSST [full adjustment, OR = 1.06, 95% *CI:* 1.02–1.10] tests. Eventually, the GAM and smooth curve fit model was conducted to confirm that the association between serum globulin and cognitive impairment was non-linear. Moreover, the inflection point was 27 g/L serum globulin based on the CERAD test and 35 g/L serum globulin based on the AF test. Finally, the interaction term between serum globulin and cognitive impairment based on the AF test indicated no significant interactions among all variables (all *p* for interaction >0.05).

**Conclusion:**

The association between serum globulin levels and cognitive impairment is non-linear. A threshold effect exists between serum globulin and cognitive impairment. Large-scale prospective clinical trials are needed to validate our findings.

## Introduction

While globalization is sweeping the world, aging is also quietly hitting the world (1). Aging aggravates the prevalence of cognitive impairment (2). The most intuitive feeling is a sharp increase in the number of patients with cognitive impairment (3). Consequences of cognitive dysfunction include memory decline, reduced social mobility, and spatial cognitive impairment (4, 5). The disease specificity of cognitive impairment has brought serious economic and social burdens to society, especially in low-income countries (6, 7). A report estimated that the number of dementia cases will reach 150 million globally by 2050 (8). This figure is also very significant in the United States (US), which is estimated to be as high as 13.8 million by 2060 (9). This will pose serious economic and social challenges (10). Effective treatment methods and interventions will be worth investigating (4). Unfortunately, the current interventions have limited efficacy (11). Early prevention including identification of risk and protective factors may be an effective pathway (12–15).

Risk and protective factors of cognitive dysfunction and beneficial diet are important research directions and breakthroughs (4, 16–18). In recent years, serum globulin as a liver function marker has been used to predict other diseases such as stroke, ulcerative colitis, atrial fibrillation, rheumatoid arthritis, nasopharyngeal carcinoma, and hepatitis C virus and now it has become a hot spot of theoretical research (19–24). The relationship between serum globulin and cognitive function is also a current research hot spot (25–28). Data showed that serum globulin is related to cognitive function (29). However, Serum ApoB activity might relate to cognitive decline rather than serum globulin (30). Another research confirmed that there was a correlation between cognitive decline and serum albumin/globulin ratio (A/G ratio) (31).

Considering that existing studies do not fully understand the association between serum globulin and cognitive function, we consulted the National Health and Nutrition Examination Survey (NHANES) data analysis from 2011 to 2012 and 2013 to 2014 to verify the association between serum globulin and cognitive impairment in older American adults. To the best of our knowledge, this is the first study to determine the association between serum globulin and cognitive impairment based on clinical public data.

## Methods

### Study population

**Figure 1:**
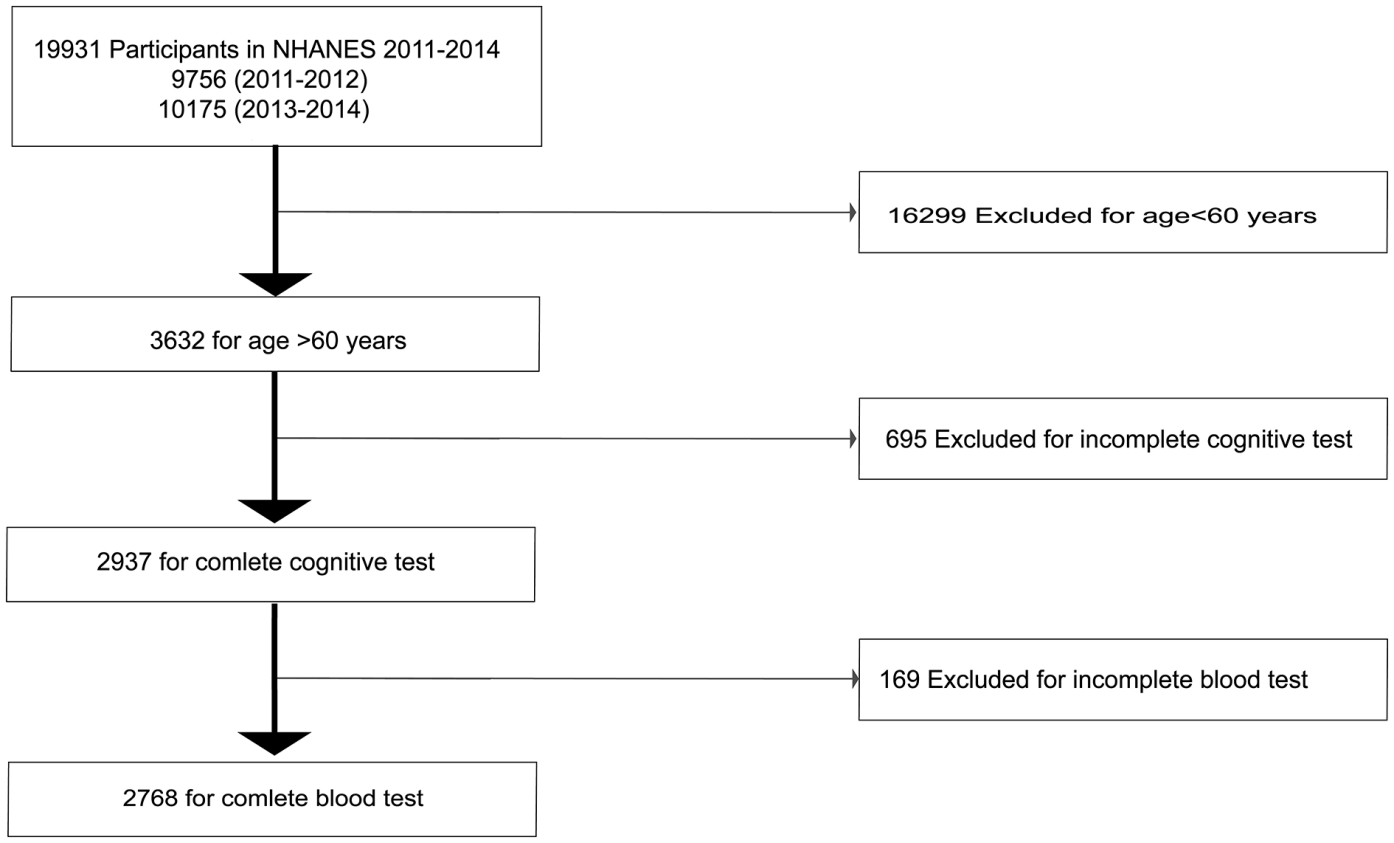
Flow chart showing the selection of study participants.

We extracted data from NHANES (2011–2012 and 2013–2014). The NHANES public database launched by the U.S. Centers for Disease Control and Prevention (CDC) is designed to evaluate the health status and nutrition level of the United States population, releasing data on a 2-year cycle (32–36). To date, thousands of secondary analyses have been performed globally based on NHANES data. Including 2011–2012 (*n* = 9,756) and 2013–2014 (*n* = 10,175), a total of 19,931 Americans participated. We had the following exclusion criteria: (1) <60 years old (*n* = 16,299), (2) inability to complete cognitive function tests (*n* = 695), (3) inability to complete blood tests (*n* = 169). Thus, 2,768 participants were eventually included in the analysis (Figure 1). In this study, written informed consent was obtained from all study participants and the Research Ethics Review Committee of the National Center for Health Statistics. The secondary analysis of the public database NHANES does not require specific informed consent. This research, with a secondary analysis of NHANES, was based on the guidelines of Strengthening the Reporting of Observation Studies in Epidemiology (STROBE) (37).

### Primary exposure

In this study, we followed the guidelines in the NHANES Laboratory/Medical Technician Procedures Manual (LPM) for detailed specimen collection and processing instructions (38). Samples that needed to be tested were tested by the Collaborative Laboratory Services Department, and the samples for testing needed to be placed under specific conditions, such as the need to be packaged in vials and stored between 2 and 8°C. The value range of serum globulin was 14–65 g/L. In sensitive analysis, serum globulin was transferred into a categorical variable by quartile.

### Outcome variable

The assessment of cognitive function, including working memory, delayed recall, and verbal fluency, was mainly conducted for American adults ≥60 years. The whole evaluation process was completed by the mobile detection center (MEC). Each participant recognized the recording quality and score of the completed examination.

The Consortium to Establish a Registry for Alzheimer’s Disease (CERAD) conducted research on new learning, recognition memory, and delayed recall. The CERAD Word Learning test (CERAD-WL) consists of three consecutive learning tests (39). During the assessment, after participants read 10 unrelated words aloud, they were instructed to recall as many words as they could from what they had just read. The total score of three trials was 30 points. The CERAD Delayed Recall (CERAD-DR) test asked participants to recall 10 words from the CERAD-WL test after completing other tests (16, 40).

Participants completed the Animal Fluency (AF) test, in which they were asked to name as many animals as possible within 1 min. Language fluency was judged during the test by the number of scores the participants named the animals (41). The operator used Digit Symbol Substitution Test (DSST) to evaluate participants’ working memory, processing speed, and continuous attention. The whole test was completed in 2 min. By copying the symbols of 133 boxes, the more correct the matching, the higher the score (42).

At present, in the published literature, the scoring criteria of cognitive impairment have not been completely unified. The dividing point of cognitive impairment is usually 25% of the total score (43). Consistent with the references, CERAD < 5, AF < 14, and DSST < 34 were considered to suffer cognitive impairment.

### Covariates

We referred to the historical literature for possible confounding factors, which mainly included three factors: sociodemographic factors, lifestyle, health status, and laboratory tests.

Sex, age, race, education, marital status, and poverty-to-income ratios were included in sociodemographic factors. Ages were divided into three groups: 60–69, 70–79, and ≥ 80 years. Race included five groups: Mexican American/Other Hispanic, Non-Hispanic White, Non-Hispanic Black, Non-Hispanic Asian, and Other. The educational levels of the subjects were classified as below high school, high school, and above high school. Marital status was divided into married/living with a partner, widowed/divorced/separated, and never married. The poverty-to-income ratio included two states: <1 and > 1.

In addition, lifestyle included alcohol consumption (12 alcoholic drinks per year), smoking habits (at least 100 cigarettes), and vigorous work activity (yes or no) (44). Health status was divided according to the history of coronary heart disease, stroke, diabetes, hypertension, and high cholesterol. Body mass index (BMI) included three statuses: <25, 25–30, and > 30 kg/m^2^. A nine-question patient health questionnaire (PHQ9) was used to assess depressive status. Depression was defined as a score greater than 10 in historical literature (45). Laboratory tests included alanine aminotransferase (ALT), aspartate aminotransferase (AST), alkaline phosphatase (ALP), gamma-glutamyl transferase (GGT), total bilirubin, total protein, albumin, blood urea nitrogen (BUN), uric acid, and creatinine.

### Statistical analysis

First, the two types of variables were expressed in different ways, in which continuous variables were described by weighted mean ± standard deviation, and differences were compared by one-way ANOVA. Conversely, weighted percentages were used to describe categorical variables and differences were compared by a chi-square test.

Second, in the current cross-sectional study, we used a weighted multivariate logistic regression model to effectively explore the association between serum globulin and cognitive impairment. Next, the model was fully adjusted in four areas: sociodemographic factors (sex, age, race, education level, marital status, and poverty-income ratio), lifestyle (alcohol consumption, smoking habit, and vigorous work activity), health status (BMI, depressive, and the history of coronary heart disease, stroke, diabetes, hypertension, and high cholesterol), and laboratory tests (ALT, AST, GGT, ALP, BUN, total bilirubin, total protein, albumin, creatinine, and uric acid). Whereas the minor model was just adjusted in three variables: sex, age, and race.

Third, we constructed generalized additive models (GAMs) and a smooth curve fit (penalty spline method) to detect any non-linear relationship between serum globulin and cognitive impairment. The linear fitting model (linear regression model) is significantly different from the non-linear fitting model (two-piecewise linear regression model) based on the *p* value of the log-likelihood ratio test <0.05. A two-piecewise linear regression model was suitable to evaluate the non-linear relationship between serum globulin and cognitive impairment. Moreover, a recursive algorithm method was used to automatically calculate threshold or inflection points.

Finally, we conducted subgroup analysis and interaction terms to verify the result. We divided each continuous variable into three groups in subgroup analysis. Furthermore, except for the subgroup variable itself, all variables were adjusted in the subgroup analysis.

Moreover, to more sensitively determine the association between serum globulin and cognitive impairment, serum globulin was transferred into a categorical variable by quartile and was assessed by *p* value for trend.

All statistical analyses were completed by R software,^1^ EmpowerStats (http://www.empowerstats.com, X&Y Solutions, Inc., United States). We employed a full-sample 4-year MEC exam weighted to ensure that the survey was representative of all older adults. A bilateral test was performed, and *p* < 0.05 confirmed a statistically significant difference.

## Results

### Characteristics of study participants

Participants from 2011 to 2014 in NHANES were included in this study, and 2,768 participants over the age of 60 met the study design and entered the final statistical analysis. The overall characteristics of all study populations are statistically analyzed in Table 1, which is the quartile of serum globulin. First, the distribution of cognitive impairment for the primary outcome measure was that in all samples, 21.13% of CERAD < 5, 21.19% of AF < 14, and 14.54% of DSST < 34. Except for three variables, namely, age, smoking history, and coronary heart disease history (*p* > 0.05), the differences of other variables in serum globulin after quartile grouping were statistically significant. First, higher levels of serum globulin were found in women aged 60–69 years old with a BMI > 30 and a history of coronary heart disease, stroke, diabetes, and hypertension. However, lower levels of serum globulin were associated with the following factors: non-Hispanic White, above high school, married/living with a partner, poverty-income ratio > 1, alcohol, BMI 25–30, and history of high cholesterol.

**Table 1:** General characteristics of participants (*n* = 2,768) stratified by serum globulin (1–4, g/L) in the NHANES 2011–2014.

Characters	Total (*n* = 2,768)	Quartiles 1 (<25; *n* = 537)	Quartiles 2 (25–28; *n* = 707)	Quartiles 3 (28–31; *n* = 689)	Quartiles 4 (>31; *n* = 835)	*p* value
Sex						0.0021
Male	46.05	44.64	50.9	45.34	41.03	
Female	53.95	55.36	49.1	54.66	58.97	
Age (years)						0.2186
60–69	56.05	55.67	58.04	53.08	56.72	
70–79	29.43	27.66	29.23	32.13	29.02	
≥80	14.52	16.67	12.73	14.79	14.26	
Race						<0.0001
Mexican American/other Hispanic	6.94	3.49	5	9.28	11.85	
Non-Hispanic White	80.22	91.81	87.99	74.87	58.94	
Non-Hispanic Black	7.96	1.8	4.29	8.3	21.32	
Non-Hispanic Asian	3.27	1.48	2.32	4.2	6.02	
Other Race	1.61	1.41	0.39	3.36	1.86	
Education						<0.0001
Less than high school	15.72	10.53	11.58	21.65	22.33	
High school	22.3	20.9	21.26	19.92	28.4	
Above high school	61.97	68.56	67.17	58.44	49.19	
Not recorded	0.02				0.08	
Marital status						<0.0001
Married/living with a partner	64.83	68.63	69.98	60.17	57	
Widowed/divorced/separated	30.74	26.87	25.79	36.69	36.91	
Never married	4.4	4.44	4.23	3.14	6.04	
Not recorded	0.02	0.05			0.05	
Poverty-income ratio						<0.0001
<1	8.37	5.39	5.33	12.03	12.94	
>1	85.41	88.67	88.56	83.56	78.31	
Not recorded	6.22	5.94	6.11	4.41	8.75	
Alcohol (12 alcoholic drinks per year)						<0.0001
Yes	72.82	78.43	75.23	69.67	65.28	
Smoked (at least 100 cigarettes)						0.3553
Yes	50.1	49.83	48.56	54.24	48.26	
Vigorous work activity						0.0159
Yes	12.9	12.18	16	12.28	9.64	
BMI (kg/m^2^)						<0.0001
<25	26	29.71	25.34	24.8	23.59	
25–30	36.17	39.75	38.58	33.45	30.79	
>30	36.36	29.97	34.13	40.22	43.85	

Characters	Total (*n* = 2,768)	Quartiles 1 (<25; *n* = 537)	Quartiles 2 (25–28; *n* = 707)	Quartiles 3 (28–31; *n* = 689)	Quartiles 4 (>31; *n* = 835)	*p* value
History of coronary heart disease						0.191
Yes	9.66	9.44	10.33	10.16	8.33	
History of stroke						<0.0001
Yes	6.52	7.09	3.61	6.73	10.13	
History of diabetes						<0.0001
Yes	18.96	18.86	14.3	17.88	27.62	
History of hypertension						<0.0001
Yes	58.35	52.9	55.94	60.15	67.2	
History of high cholesterol						0.0498
Yes	57.49	59.94	54.86	58.65	57.16	
Depressive (>10)	6.98	6.36	4.86	7.1	10.98	0.0001
ALT (U/L)	22.13 ± 11.43	21.54 ± 7.66	22.13 ± 11.14	21.30 ± 9.34	23.82 ± 16.68	0.0006
AST (U/L)	25.02 ± 9.72	24.17 ± 6.48	24.33 ± 6.69	24.71 ± 8.35	27.58 ± 16.14	<0.0001
ALP (U/L)	67.50 ± 22.16	62.51 ± 18.12	64.26 ± 17.60	69.55 ± 21.26	76.79 ± 29.88	<0.0001
GGT (U/L).	25.58 ± 30.52	23.18 ± 20.94	21.61 ± 16.42	26.51 ± 28.71	33.91 ± 51.32	<0.0001
Total protein (g/L)	69.38 ± 4.56	65.12 ± 3.04	68.56 ± 2.67	70.69 ± 2.96	74.71 ± 4.01	<0.0001
Albumin (g/L)	42.10 ± 2.91	42.81 ± 2.57	42.51 ± 2.57	41.80 ± 2.96	40.89 ± 3.32	<0.0001
Total bilirubin (μmol/L)	11.76 ± 4.72	12.00 ± 4.43	11.91 ± 4.89	11.42 ± 3.96	11.60 ± 5.50	0.0884
Creatinine (μmol/L)	87.84 ± 51.49	86.54 ± 48.23	86.85 ± 31.92	85.08 ± 28.02	94.17 ± 87.58	0.0119
BUN (mmol/L)	5.81 ± 2.44	5.74 ± 2.10	5.78 ± 2.30	5.82 ± 2.29	5.91 ± 3.11	0.6745
Uric acid (μmol/L)	333.59 ± 85.02	315.21 ± 78.48	331.66 ± 81.53	337.81 ± 82.57	355.72 ± 95.07	
CERAD (<5)	21.13	19.7	19.46	25.06	21.24	0.0444
AF (<14)	21.19	15.56	18.38	23.67	30.13	<0.0001
DSST (<34)	14.54	8.03	11.01	17.93	24.75	<0.0001

Mean ± SD for: ALT (U/L), AST (U/L), ALP (U/L), GGT (U/L), total bilirubin (μmol/L), total protein (g/L), albumin (g/L), BUN (mmol/L), creatinine (μmol/L), and uric acid (μmol/L). *p* value was calculated by one-way ANOVA.

% for: sex, age, race, education, marital status, poverty-income ratio, alcohol consumption, smoking status, vigorous work activity, BMI (kg/m^2^), depression, history of coronary heart disease, history of stroke, history of diabetes, history of hypertension, history of high cholesterol, CERAD, and AF, DSST. *p* value was calculated by a weighted chi-square test.

### Association between serum globulin and cognitive impairment

The association between serum globulin and cognitive impairment based on the CERAD, AF, and DSST tests is shown in Table 2. Three multivariate logistical regression models were applied to evaluate the association between serum globulin and cognitive impairment: model 1 (non-adjusted model), model 2 (minor adjusted model), and model 3 (fully adjusted model).

**Table 2:** Associations between serum globulin (g/L) and cognitive impairment (CERAD <5, AF <14, and DSST <34; *n* = 2,768), NHANES 2011–2014.

	Model 1 OR (95% CI), *p*	Model 2 OR (95% CI), *p*	Model 3 OR (95% CI), *p*
CERAD < 5			
Globulin (g/L)	1.00 (0.99, 1.02) 0.6381	1.00 (0.98, 1.02) 0.7266	1.01 (0.97, 1.04) 0.7312
Quintiles of globulin			
Q1 (<25)	1	1	1
Q2 (25–28)	1.09 (0.84, 1.42) 0.5097	1.15 (0.87, 1.52) 0.3173	1.31 (0.94, 1.82) 0.1149
Q3 (28–31)	1.21 (0.93, 1.57) 0.1535	1.24 (0.93, 1.64) 0.1373	1.30 (0.87, 1.95) 0.2067
Q4 (>31)	1.11 (0.86, 1.44) 0.4025	1.12 (0.84, 1.49) 0.4285	1.24 (0.69, 2.23) 0.4650
*p* for trend	0.943	0.995	0.501
AF < 14			
Globulin (g/L)	1.06 (1.04, 1.07) <0.0001	1.03 (1.01, 1.05) 0.0020	1.05 (1.01, 1.08) 0.0075
Quintiles of globulin			
Q1 (<25)	1	1	1
Q2 (25–28)	1.26 (0.96, 1.64) 0.0926	1.19 (0.90, 1.58) 0.2093	1.31 (0.95, 1.82) 0.1000
Q3 (28–31)	1.58 (1.22, 2.06) 0.0006	1.32 (1.00, 1.74) 0.0518	1.48 (1.01, 2.18) 0.0463
Q4 (>31)	2.29 (1.79, 2.94) <0.0001	1.68 (1.28, 2.21) 0.0002	1.98 (1.15, 3.41) 0.0134
*p* for trend	<0.001	<0.001	0.12
DSST < 34			
Globulin (g/L)	1.09 (1.07, 1.11) <0.0001	1.07 (1.05, 1.09) <0.0001	1.06 (1.02, 1.10) 0.0040
Quintiles of globulin			
Q1 (<25)	1	1	1
Q2 (25–28)	1.59 (1.17, 2.17) 0.0031	1.51 (1.09, 2.10) 0.0141	1.46 (0.97, 2.18) 0.0700
Q3 (28–31)	2.49 (1.85, 3.36) <0.0001	2.06 (1.50, 2.85) <0.0001	1.59 (1.00, 2.54) 0.0501
Q4 (>31)	3.60 (2.71, 4.79) <0.0001	2.65 (1.93, 3.64) <0.0001	1.63 (0.85, 3.10) 0.1402
*p* for trend	<0.001	<0.001	0.711

Model 1: non-adjusted model; Model 2: adjust for age, race, sex; Model 3: adjust for age, race, sex, education, marital status, poverty-income ratio, alcohol consumption, smoking status, vigorous work activity, BMI, history of coronary heart disease, diabetes, stroke, hypertension, high cholesterol, depressive, ALT (U/L), AST (U/L), ALP (U/L), GGT (U/L), total bilirubin (μmol/L), total protein (g/L), Albumin (g/L), BUN (mmol/L), uric acid (μmol/L), and creatinine (μmol/L).

First, a statistically significant difference was not present in each model between serum globulin and cognitive impairment based on the CERAD test.

Second, a statistically significant difference was shown in each model between serum globulin and elevated risk of cognitive impairment based on the AF test. In the non-adjust model, the OR with 95% *CI* was 1.06 (1.04, 1.07), and AF in Q3-Q4 [Q3: OR = 1.58, 95% *CI:* 1.22–2.06, Q4: OR = 2.29, 95% *CI:* 1.79–2.94]. After adjusting for age, sex, and race, statistically significant differences existed between serum globulin and elevated risk of cognitive impairment [OR = 1.03, 95% *CI:* 1.01–1.05] and AF in Q4 [Q4: OR = 1.68, 95% *CI:* 1.28–2.21]. A fully adjusted model also showed a statistically significant difference between serum globulin and cognitive impairment [OR = 1.05, 95% *CI:* 1.01–1.08].

Third, statistically significant differences were identified in each model between serum globulin and elevated risk of cognitive impairment based on the DSST test [full adjustment, OR = 1.06, 95% *CI:* 1.02–1.10].

### Identification of non-linear relationship

**Figure 2:**
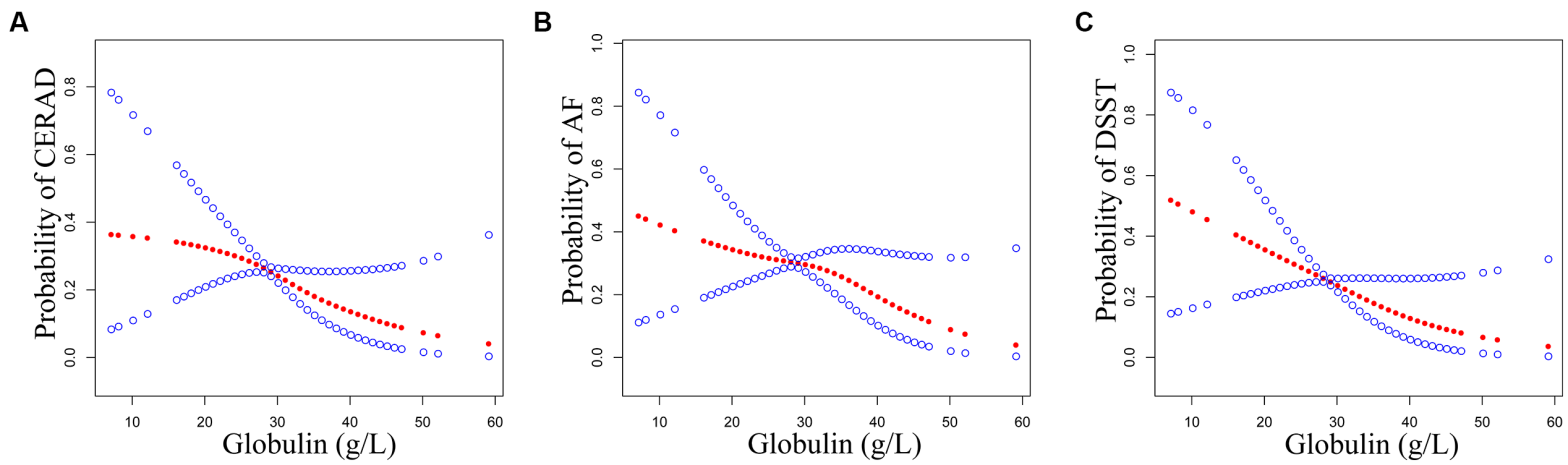
Association between serum globulin (g/L) and cognitive impairment. The probability of CERAD **(A)**, AF **(B)**, and DSST **(C)** represent the probability of cognitive impairment by GAM and smooth curve fit. The red points show a smooth curve fitting line and the blue points show a 95% confidence interval. The relationship adjusted for age, race, sex, education, marital status, poverty-income ratio, alcohol consumption, smoking status, vigorous work activity, BMI, history of coronary heart disease, diabetes, stroke, hypertension, high cholesterol, depression, ALT (U/L), AST (U/L), ALP (U/L), GGT (U/L), total bilirubin (μmol/L), total protein (g/L), Albumin (g/L), BUN (mmol/L), uric acid (μmol/L), and creatinine (μmol/L).

The GAM was conducted to assess whether there was a non-linear relationship between serum globulin and cognitive impairment (Figure 2). After full adjustment, results showed that the association between serum globulin and cognitive impairment was non-linear based on the CERAD and AF tests.

Adopting a weighted two-piecewise linear regression model and a recursive algorithm method, confirmed the turning point was 27 g/L based on the CERAD test (Table 3). On the left of the turning point or less than 27 g/L, the OR value and 95% *CI* were 1.07 and (1.00, 1.14; *p* = 0.0392), respectively. On the right of the inflection point or more than 27 g/L, the OR value and 95% *CI* were 0.98 and (0.94, 1.02), respectively.

**Table 3:** Nonlinearity addressing by weighted two-piecewise linear model based on CERAD and AF tests.

Outcome	CERAD log_2_ transform OR (95% CI), *p*	Outcome	AF log_2_ transform OR (95% CI), *p*
Fitting by a weighted linear regression model	1.01 (0.97, 1.04) 0.7312	Fitting by a weighted linear regression model	1.05 (1.01, 1.08) 0.0075
Fitting by a weighted two-piecewise logistic regression model			
Inflection point	27	Inflection point	35
< 27	1.07 (1.00, 1.14) 0.0392	< 35	1.07 (1.03, 1.11) 0.0004
> 27	0.98 (0.94, 1.02) 0.4072	> 35	0.96 (0.90, 1.03) 0.3133
Log-likelihood ratio test	0.02	Log-likelihood ratio test	0.007

The relationship adjusted for age, race, sex, education, marital status, poverty-income ratio, alcohol consumption, smoking status, vigorous work activity, BMI, history of coronary heart disease, diabetes, stroke, hypertension, high cholesterol, depression, ALT (U/L), AST (U/L), ALP (U/L), GGT (U/L), total bilirubin (μmol/L), total protein (g/L), Albumin (g/L), BUN (mmol/L), uric acid (μmol/L), and creatinine (μmol/L).

Using a weighted two-piecewise linear regression model and a recursive algorithm method, data indicated that the inflection point was 35 g/L based on the AF test (Table 3). On the left of the turning point, OR with 95% *CI* were 1.07 and (1.03, 1.11; *p* = 0.0004). On the right of the turning point or more than 35 g/L, OR value and 95% *CI* were 0.96 and (0.90, 1.03), respectively.

### Subgroup analyses outcomes

Table 4 presents the subgroup analysis and interaction results based on the CERAD, AF, and DSST tests.

**Table 4:** Subgroup analysis of all variables and interaction tests.

	CERAD < 5		AF <14		DSST <34	
	OR (95% CI), *p*	*p* value of interaction	OR (95% CI), *p*	*p* value of interaction	OR (95% CI), *p*	*p* value of interaction
Sex		0.7520		0.0831		0.9344
Male	1.00 (0.95, 1.05) 0.9136		1.04 (0.99, 1.09) 0.0942		1.06 (1.01, 1.12) 0.0248	
Female	1.01 (0.96, 1.07) 0.6399		1.06 (1.01, 1.11) 0.0200		1.05 (0.99, 1.11) 0.1366	
Age (years)		0.1943		0.5520		0.0731
60–69	1.04 (0.99, 1.10) 0.1072		1.07 (1.02, 1.12) 0.0075		1.05 (0.99, 1.11) 0.1241	
70–79	1.02 (0.95, 1.09) 0.6097		1.02 (0.96, 1.09) 0.4934		1.09 (1.01, 1.18) 0.0246	
≥80	0.92 (0.85, 0.99) 0.0246		1.03 (0.95, 1.11) 0.4930		1.06 (0.97, 1.16) 0.1797	
Race		0.2158		0.1726		0.0567
Mexican American/other Hispanic	1.04 (0.96, 1.13) 0.3514		1.11 (1.03, 1.21) 0.0095		1.11 (1.01, 1.22) 0.0229	
Non-Hispanic White	1.00 (0.95, 1.06) 0.9375		1.06 (1.00, 1.11) 0.0541		1.10 (1.03, 1.17) 0.0057	
Non-Hispanic Black	1.00 (0.93, 1.08) 0.9676		1.01 (0.95, 1.08) 0.7222		1.01 (0.93, 1.08) 0.8808	
Non-Hispanic Asian	0.86 (0.70, 1.06) 0.1657		1.15 (1.00, 1.31) 0.0505		0.88 (0.63, 1.22) 0.4458	
Other race	0.00 (0.00, Inf) 0.9999		0.03 (0.00, Inf) 1.0000		30.05 (0.00, Inf) 1.0000	
Education		0.0678		0.2566		0.0795
Less than high school	1.01 (0.95, 1.08) 0.6792		1.03 (0.97, 1.09) 0.3707		1.10 (1.03, 1.18) 0.0045	
High school	0.98 (0.91, 1.05) 0.5563		1.05 (0.98, 1.13) 0.1687		0.97 (0.90, 1.05) 0.4817	
Above high school	1.01 (0.96, 1.07) 0.6300		1.09 (1.03, 1.15) 0.0018		1.10 (1.02, 1.19) 0.0150	
Marital status		0.7011		0.2534		0.8970
Married/living with a partner	1.00 (0.95, 1.05) 0.9625		1.04 (0.99, 1.09) 0.1466		1.04 (0.98, 1.10) 0.2116	
Widowed/divorced/separated	1.00 (0.95, 1.06) 0.8875		1.08 (1.03, 1.14) 0.0037		1.09 (1.02, 1.15) 0.0100	
Never married	1.15 (0.96, 1.39) 0.1370		0.80 (0.65, 0.99) 0.0399		0.96 (0.76, 1.22) 0.7478	
Poverty-income ratio		0.6168		0.8312		0.5524
<1	0.93 (0.85, 1.02) 0.1147		1.01 (0.93, 1.10) 0.8418		1.06 (0.97, 1.17) 0.1977	
>1	1.01 (0.97 1.06) 0.5184		1.06 (1.02, 1.10) 0.0069		1.06 (1.01, 1.11) 0.0227	
Not recorded	1.00 (0.85, 1.17) 0.9911		1.04 (0.91, 1.20) 0.5298		1.08 (0.90, 1.31) 0.4023	
Alcohol (12 alcoholic drinks per year)		0.0065		0.9875		0.4077
Yes	1.03 (0.99, 1.08) 0.1377		1.05 (1.01, 1.09) 0.0234		1.05 (1.00, 1.10) 0.0468	
No	0.95 (0.89, 1.01) 0.1010		1.05 (0.99, 1.11) 0.1107		1.06 (0.99, 1.14) 0.0847	
Smoking status		0.0174		0.6608		0.3638
Yes	1.03 (0.98, 1.08) 0.2390		1.05 (1.01, 1.10) 0.0294		1.07 (1.02, 1.13) 0.0118	
No	0.98 (0.93, 1.03) 0.3928		1.05 (0.99, 1.10) 0.0853		1.05 (0.98, 1.11) 0.1551	
Vigorous work activity		0.5468		0.3027		0.6181
Yes	0.91 (0.79, 1.05) 0.1902		1.11 (0.97, 1.26) 0.1216		1.06 (0.87, 1.29) 0.5524	
No	1.01 (0.97, 1.05) 0.5479		1.04 (1.00, 1.08) 0.0256		1.06 (1.02, 1.10) 0.0076	
BMI (kg/m^2^)		0.4885		0.9796		0.2832
<25	1.00 (0.93, 1.08) 0.9524		1.02 (0.96, 1.10) 0.4826		1.01 (0.93, 1.10) 0.8661	
25–30	1.01 (0.95, 1.07) 0.8336		1.06 (1.00, 1.13) 0.0578		1.03 (0.96, 1.12) 0.4101	
>30	1.00 (0.95, 1.06) 0.8841		1.06 (1.01, 1.12) 0.0243		1.09 (1.03, 1.16) 0.0061	
Coronary heart disease		0.4671		0.9795		0.3984
Yes	1.06 (0.92, 1.22) 0.4338		1.08 (0.94, 1.25) 0.2748		1.13 (0.95, 1.35) 0.1646	
No	1.01 (0.97, 1.04) 0.7596		1.05 (1.01, 1.09) 0.0080		1.05 (1.01, 1.10) 0.0182	
Not recorded	1.00 (0.00, Inf) 1.0000		2.06 (0.00, Inf) 1.0000		1.00 (0.00, Inf) 1.0000	
Stroke		0.6883		0.3734		0.9590
Yes	0.91 (0.79, 1.06) 0.2347		1.02 (0.88, 1.18) 0.8042		1.01 (0.86, 1.17) 0.9365	
No	1.01 (0.97, 1.04) 0.7379		1.05 (1.01, 1.08) 0.0086		1.06 (1.02, 1.11) 0.0061	
Diabetes		0.6855		0.0836		0.0969
Yes	1.07 (1.00, 1.15) 0.0472		1.07 (1.00, 1.15) 0.0374		1.08 (1.00, 1.16) 0.0488	
No	0.99 (0.95, 1.03) 0.6424		1.04 (1.00, 1.09) 0.0412		1.05 (1.00, 1.11) 0.0443	
Not recorded	1.01 (0.78, 1.31) 0.9379		1.06 (0.82, 1.38) 0.6408		1.02 (0.61, 1.72) 0.9372	
Hypertension		0.3908		0.8641		0.3865
Yes	1.00 (0.96, 1.05) 0.8410		1.04 (0.99, 1.08) 0.0935		1.05 (1.00, 1.10) 0.0304	
No	1.01 (0.95, 1.08) 0.7781		1.07 (1.01, 1.14) 0.0281		1.06 (0.98, 1.15) 0.1666	
High cholesterol		0.9164		0.1109		0.6929
Yes	1.02 (0.97, 1.07) 0.4390		1.05 (1.00, 1.10) 0.0313		1.09 (1.03, 1.15) 0.0040	
No	1.00 (0.95, 1.06) 0.9393		1.06 (1.01, 1.11) 0.0304		1.03 (0.98, 1.10) 0.2561	
Not recorded	0.00 (0.00, Inf) 0.9998		0.00 (0.00, Inf) 0.9999		0.06 (0.00, Inf) 1.0000	
Depression		0.5030		0.4279		0.6631
Yes	1.00 (0.96, 1.04) 0.8537		1.04 (1.00, 1.08) 0.0268		1.06 (1.01, 1.10) 0.0137	
No	1.03 (0.92, 1.15) 0.5742		1.06 (0.95, 1.18) 0.2778		1.02 (0.90, 1.14) 0.8011	
ALT (U/L)		0.8356		0.0743		0.7686
5–16	0.97 (0.91, 1.03) 0.3212		1.09 (1.03, 1.16) 0.0063		1.06 (0.99, 1.14) 0.0979	
17–22	1.05 (0.98, 1.12) 0.1423		1.01 (0.95, 1.08) 0.6769		1.07 (0.99, 1.15) 0.0863	
23–228	0.99 (0.93, 1.06) 0.8431		1.05 (0.99, 1.11) 0.1019		1.03 (0.96, 1.10) 0.4252	
AST (U/L)		0.2914		0.8673		0.0358
9–20	0.95 (0.88, 1.02) 0.1475		1.06 (0.99, 1.14) 0.0784		1.03 (0.95, 1.12) 0.5104	
21–25	1.03 (0.97, 1.10) 0.3603		1.02 (0.96, 1.09) 0.4597		1.07 (0.99, 1.16) 0.0734	
26–220	1.00 (0.94, 1.06) 0.9932		1.04 (0.98, 1.09) 0.2052		1.05 (0.99, 1.12) 0.1279	
GGT (U/L)		0.5691		0.8454		0.2023
4–15	1.04 (0.97, 1.11) 0.2266		1.11 (1.04, 1.19) 0.0021		1.14 (1.05, 1.23) 0.0009	
16–24	0.99 (0.93, 1.06) 0.7950		1.04 (0.97, 1.11) 0.2577		1.04 (0.96, 1.12) 0.3444	
25–1,197	0.99 (0.93, 1.05) 0.7194		1.03 (0.98, 1.09) 0.2674		1.01 (0.95, 1.08) 0.6781	
Albumin (g/L)		0.3568		0.1653		0.1073
21–40	1.00 (0.92, 1.09) 0.9350		1.07 (0.99, 1.16) 0.1066		1.16 (1.05, 1.28) 0.0046	
41–42	0.99 (0.67, 1.45) 0.9466		0.84 (0.58, 1.22) 0.3706		1.33 (0.85, 2.08) 0.2093	
43–54	0.99 (0.90, 1.09) 0.8379		1.00 (0.91, 1.10) 0.9924		1.05 (0.94, 1.17) 0.4144	
ALP (U/L)		0.1517		0.8475		0.3536
14–58	1.00 (0.94, 1.08) 0.9180		1.05 (0.98, 1.12) 0.1454		1.03 (0.95, 1.11) 0.5326	
59–74	1.00 (0.93, 1.07) 0.9833		1.11 (1.04, 1.18) 0.0017		1.06 (0.98, 1.15) 0.1244	
75–336	1.01 (0.96, 1.07) 0.6695		1.03 (0.97, 1.08) 0.3189		1.08 (1.01, 1.15) 0.0173	
BUN (mmol/L)		0.6675		0.4950		0.5258
1.07–4.28	0.99 (0.92, 1.06) 0.6899		1.10 (1.03, 1.18) 0.0044		1.06 (0.98, 1.15) 0.1324	
4.64–5.71	1.02 (0.96, 1.10) 0.4985		1.08 (1.01, 1.15) 0.0220		1.04 (0.96, 1.12) 0.3482	
6.07–33.92	1.00 (0.95, 1.06) 0.9070		1.01 (0.95, 1.06) 0.7875		1.07 (1.00, 1.13) 0.0454	
Uric acid (μmol/L)		0.0300		0.0949		0.3157
65.4–291.5	0.98 (0.92, 1.05) 0.5959		1.09 (1.03, 1.16) 0.0059		1.06 (0.99, 1.14) 0.1031	
297.4–362.8	1.04 (0.98, 1.11) 0.1841		1.09 (1.03, 1.16) 0.0058		1.05 (0.97, 1.13) 0.2350	
368.8–701.9	0.98 (0.92, 1.04) 0.5164		0.97 (0.92, 1.03) 0.3497		1.05 (0.99, 1.13) 0.1281	
Cre (U/L)		0.0070		0.4761		0.2468
37.13–72.49	1.02 (0.95, 1.09) 0.5679		1.08 (1.01, 1.15) 0.0249		1.04 (0.96, 1.13) 0.2857	
73.37–91.94	1.04 (0.97, 1.11) 0.3138		1.06 (0.99, 1.12) 0.0868		1.10 (1.02, 1.19) 0.0098	
92.82–1539.04	0.97 (0.92, 1.03) 0.3630		1.03 (0.97, 1.08) 0.3487		1.05 (0.99, 1.12) 0.1108	
Total bilirubin (μmol/L)		0.2539		0.9277		0.7501
1.71–8.55	0.97 (0.91, 1.03) 0.2755		1.07 (1.01, 1.13) 0.0283		1.04 (0.97, 1.11) 0.2801	
10.26–10.26	1.00 (0.92, 1.09) 0.9647		1.07 (0.98, 1.16) 0.1362		1.06 (0.96, 1.17) 0.2206	
11.97–66.69	1.02 (0.97, 1.08) 0.4426		1.03 (0.98, 1.09) 0.1878		1.08 (1.01, 1.15) 0.0218	
Total protein (g/L)		0.1363		0.0625		<0.0001
53–67	1.11 (1.01, 1.21) 0.0240		0.99 (0.90, 1.07) 0.7637		1.21 (1.07, 1.38) 0.0024	
68–71	1.00 (0.85, 1.17) 0.9623		1.04 (0.90, 1.21) 0.5693		0.96 (0.79, 1.17) 0.6887	
72–95	0.97 (0.92, 1.02) 0.2539		0.99 (0.95, 1.04) 0.8145		1.06 (1.00, 1.11) 0.0367	

Each continuous variable was divided into three groups according to its value. Each subgroup analysis adjusted for all the founders except the subgroup factor itself. Potential interactions between serum globulin and cognitive impairment were tested by a likelihood ratio test.

First, interaction term results revealed a significant difference for smokers, alcohol users, and participants that reported high creatinine between serum globulin and cognitive impairment based on the CERAD test (all *p* for interaction <0.05). Subgroup analysis terms based on the CERAD test suggested that participants aged ≥80 years, those with diabetes, and 53–67 g/L total protein levels were associated with an increased risk of cognitive impairment (all *p* < 0.05).

Second, the interaction term between serum globulin and cognitive impairment based on the AF test indicated no significant interactions among all variables (all *p* for interaction >0.05). Moreover, subgroup analyses based on the AF test confirmed that participants who were aged 60–69 years old, women, or Mexican American/other Hispanic, educated above high school, widowed/divorced/separated, and never married, those with a poverty-income ratio > 1, those who consumed alcohol, performed no vigorous work activity, had no history of coronary heart disease or stroke, had diabetes, high cholesterol levels, depression, or those who had 4–15 U/L GGT, 59–74 U/L ALP, 1.07–5.71 mmol/L BUN, 65.4–362.8 μmol/L uric acid, 37.13–72.49 U/L creatinine, and 1.71–8.55 μmol/L total bilirubin had a significantly increased risk of cognitive impairment (all *p* < 0.05).

Third, interaction terms based on a DSST test between serum globulin and cognitive impairment were significant for total protein (*p* for interaction <0.05). Subgroup analysis terms based on the DSST test revealed that participants who were men, 70–79 years old, Mexican American/other Hispanic, Non-Hispanic White, educated to less than high school, educated above high school, widowed/divorced/separated, poverty-income ratio > 1, consumed alcohol, smoked, performed no vigorous work activity, had a BMI >30, had no coronary heart disease, had no stroke, had diabetes, had high cholesterol, had depression, or had 4–15 U/L GGT, 21–40 g/L albumin, 75–336 U/L ALP, 25–1,197 U/L GGT, 6.07–33.92 mmol/L BUN, 73.37–91.94 U/L creatinine, 11.97–66.69 μmol/L total bilirubin, 72–95 g/L total protein, and 53–67 g/L total protein had a significantly increased risk of cognitive impairment (all *p* < 0.05).

## Discussion

To the best of our knowledge, this is the first study to use the public sample data from 2011 to 2014 in the NHANES database to understand the association between serum globulin and cognitive impairment. At the same time, this study also conducted a beneficial exploration of the correlation between liver function and cognitive function. After adjusting for all possible confounding factors, serum globulin was associated with an elevated risk of cognitive impairment in the AF and DSST tests. Moreover, we used GAM and the smooth curve fit model to verify that this association between serum globulin and cognitive impairment is non-linear. There is an obvious serum globulin threshold of 27 g/L based on the CERAD test and 35 g/L based on the AF test. Our study differs from most previous studies in that we are the first to demonstrate the association between serum globulin and cognitive impairment.

In 2018, Frith et al. applied NHANES data to confirm the effect of physical activity on cognitive function, and their results revealed that an elevated gamma gap existed in the relationship. Gamma gaps indicate high serum globulin concentrations. All data indirectly confirmed that globulin proteins may correlate with cognitive function (46). Another study demonstrated that lower serum globulin and a higher albumin/globulin ratio were associated with increased gray matter volume in the olfactory cortex and parahippocampal gyrus (29). Our results are consistent with those of previous studies. Maeda et al. reported that the serum albumin/globulin ratio was correlated with cognitive function in 1,827 Japanese older adults. However, serum albumin and globulin levels were not associated with cognitive function (31). Zhao et al. confirmed whether cognitive function was correlated to liver function. Data demonstrated that only serum ApoB activity, rather than serum globulin levels, may be associated with cognitive deficits (30). Another study revealed that the A/G ratio was the only factor that significantly lowered cognitive decline risk (27).

Compared with the recent literature, the present study has some main advantages. First, the large sample size may be more statistically significant in exploring the effect of serum globulin on cognitive impairment. This study included 2,768 older adults to better study the effect of serum globulin levels on cognitive function. Second, this study eliminated different categories of missing data to decrease the potential impact of missing data. Third, as many confounding factors as possible were included, including the participants’ chronic disease history and depression status, and three mainstream experiments were adopted to evaluate cognitive function. Finally, the non-linear relationship between serum globulin levels and cognitive impairment was verified using a smooth curve fitting model, and the serum globulin threshold level was confirmed via threshold analysis.

However, this study had some limitations, which might have affected its outcomes. First, a causal relationship between serum globulin and cognitive impairment is difficult to distinguish, which is determined via the inherent characteristics of cross-sectional studies. Second, the NHANES study population was limited to Americans, so the generalizability of our results is geographically limited. Third, some older adults with potential cognitive impairment might have been excluded due to their inability to complete the interviews for cognitive function assessment.

## Conclusion

The association between serum globulin and cognitive impairment is non-linear. A threshold effect was confirmed between serum globulin levels and cognitive impairment. Larger prospective clinical trials such as cohort studies and Mendelian analysis based on the association between serum globulin and cognitive impairment are needed in the future to confirm the current results.

## Data availability statement

The original contributions presented in the study are included in the article/supplementary material, further inquiries can be directed to the corresponding author.

## Ethics statement

The studies involving humans were approved by Research Ethics Review Committee of the National Center for Health Statistics. The studies were conducted in accordance with the local legislation and institutional requirements. The participants provided their written informed consent to participate in this study. The manuscript presents research on animals that do not require ethical approval for their study.

## Author contributions

JH and ZL planned and executed the studies, data analysis, and drafted the manuscript. RL processed and analysis the data. HZ and DH have made great efforts to revise the manuscript. WL conceived the item. JW facilitated design and analysis of the experiments. All authors contributed to the article and approved the submitted version.

## Funding

The study was funded by the National Natural Science Foundation of China (81471197 and 81070950).

## Conflict of interest

The authors declare that the research was conducted in the absence of any commercial or financial relationships that could be construed as a potential conflict of interest.

## Publisher’s note

All claims expressed in this article are solely those of the authors and do not necessarily represent those of their affiliated organizations, or those of the publisher, the editors and the reviewers. Any product that may be evaluated in this article, or claim that may be made by its manufacturer, is not guaranteed or endorsed by the publisher.

## Footnotes

^1^ http://www.R-project.org
